# Glucocorticoid-induced leucine zipper (GILZ) in immuno suppression: master regulator or bystander?

**DOI:** 10.18632/oncotarget.6197

**Published:** 2015-10-20

**Authors:** Jessica Hoppstädter, Alexandra K. Kiemer

**Affiliations:** ^1^ Department of Pharmacy, Pharmaceutical Biology, Saarland University, Saarbrücken, Germany

**Keywords:** inflammation, macrophage, lipopolysaccharide, glucocorticoids, mouse models

## Abstract

Induction of glucocorticoid-induced leucine zipper (GILZ) by glucocorticoids has been reported to be essential for their anti-inflammatory actions. At the same time, GILZ is actively downregulated under inflammatory conditions, resulting in an enhanced pro-inflammatory response. Two papers published in the recent past showed elevated GILZ expression in the late stage of an inflammation. Still, the manuscripts suggest seemingly contradictory roles of endogenous GILZ: one of them suggested compensatory actions by elevated corticosterone levels in GILZ knockout mice, while our own manuscript showed a distinct phenotype upon GILZ knockout *in vivo*. Herein, we discuss the role of GILZ in inflammation with a special focus on the influence of endogenous GILZ on macrophage responses and suggest a cell-type specific action of GILZ as an explanation for the conflicting results as presented in recent reports.

## INTRODUCTION

The inflammatory response protects the body against infections and injuries, but its deregulation can be detrimental to the host. Endogenous pathways activated during the late stage of defense reactions can counter regulate inflammation and promote resolution.

Disruption of such counter-regulatory mechanisms may result in exacerbated inflammatory responses. On the other hand, excessive activation of the anti-inflammatory cascades might lead to severe immunosuppression [1, 2].

Both states can be observed during sepsis, which is commonly caused by a widespread bacterial infection. Pro- and anti-inflammatory processes are initiated rapidly after the onset of sepsis, although the hyper-inflammatory response is predominant in the first phase of the disease. The extent of inflammation can be influenced by many factors including pathogen virulence, bacterial load, age, and comorbidities. In this early phase of sepsis, deaths are generally due to cardiovascular collapse and multiple organ failure [1].

In order to limit tissue damage, the hyper-inflammatory phase is followed by compensatory anti-inflammatory responses. When poorly timed or too excessive, immunosuppression might leave the host vulnerable towards secondary infections, which represent a main cause of death in this late stage of the disease. Patients that develop impaired immunity can either be unable to eradicate the primary infection or develop secondary hospital-acquired infections, as indicated by the rise of positive blood cultures for typically opportunistic bacteria [1, 3–6]. On the cellular level, macrophages are major contributors to both inflammation and immunosuppression [4, 7].

## THE ROLE OF MACROPHAGES IN THE COURSE OF INFLAMMATION

Macrophages are a highly diverse subtype of immune cells. Their functionally distinct phenotypes play key roles in acute and chronic inﬂammation as well as the resolution of inﬂammation and ﬁbrosis [8–11].

When pathogens invade tissues, circulating monocytes are recruited and differentiate into macrophages, which then co-exist with resident tissue macrophages [12]. Subsequently, pathogen- or danger-associated molecular patterns are detected *via* pattern recognition receptors, such as toll-like receptors (TLRs) [13].

In general, macrophages are polarized towards an inflammatory phenotype in the early stage of bacterial infections. With reference to Th1/Th2 polarization, two distinct macrophage phenotypes have been suggested: the classically activated or inflammatory (M1) macrophage and the alternatively activated (M2) macrophage. However, there are also numerous dynamic intermediates [14, 15]. The TLR4 agonist lipopolysaccharide (LPS) and the Th1 cytokine IFN-γ polarize macrophages towards the M1 phenotype associated with the production of large amounts of pro-inflammatory mediators, such as tumor-necrosis factor (TNF)-α, nitric oxide, IL-12, and IL-23. Thus, M1 macrophages promote pathogen clearance and antigen-specific Th1 and Th17 responses. In contrast, exposure of macrophages to the Th2 cytokines IL-4 or IL-10 induces an M2 phenotype characterized by the production of high levels of IL-10 and IL-1 receptor antagonist and low expression of IL-12. M2 macrophages are more heterogeneous, but generally play a role in Th2 responses, such as killing or encapsulation of extracellular parasites, resolving type 1 inflammation, and tissue repair and remodeling. M2 macrophages are not only important in immune regulation, but also promote tumor progression [8–11].

Recent studies have described another type of macrophage, the resolving macrophage (Mres). A major function of macrophages during the resolution of inflammation is efferocytosis, i.e. phagocytosis and clearance, of apoptotic neutrophils. Unlike phagocytosis of pathogens, which usually induces proinflammatory responses, efferocytosis leads to reprogramming and immune silencing [16, 17]. When macrophages encounter apoptotic neutrophils and start to engulf them, the macrophages switch to an M2-like phenotype that is anti-inflammatory, highly phagocytic, and involved in tissue repair, but can also contribute to fibrosis [17]. As the uptake of apoptotic leukocytes progresses, macrophages undergo another phenotypic switch into Mres. These macrophages reduce the expression of pro-fibrotic factors and display reduced phagocytosis of extracellular particles including apoptotic cells. Anti-inflammatory and pro-resolving mediators, such as glucocorticoids, IL-4, IL-10, PPAR-γ ligands, lipoxins, and resolvins can further modulate efferocytosis. This modulation can enhance the immune silencing and departure of Mres to the lymphatic system, where they promote the termination of the acquired immune response. Mres that remain in the tissue express high levels of anti-inflammatory, anti-fibrotic, and anti-oxidant mediators in order to limit tissue damage and fibrosis [17–20].

Functional skewing of macrophages is also evident during sepsis. Acute and prolonged stimulation of macrophages and monocytes by LPS or other TLR ligands results in excessive inflammation, followed by a hyporesponsive state termed endotoxin or LPS tolerance in later stages of the disease. LPS-tolerant macrophages are characterized by a reduced secretion of pro-inflammatory cytokines, upregulation of anti-inflammatory genes, and increased phagocytosis as well as wound healing properties and are therefore considered as an M2-like population [4, 5, 21]. Monocytes and macrophages are the principal cells responsible for the induction of LPS tolerance *in vivo*. There are still many unknown aspects regarding the molecular mechanisms underlying LPS tolerance, although defects in TLR4 signaling have been suggested to be involved [4, 5].

We [22] and others [23] recently reported elevated levels of glucocorticoid-induced leucine zipper (GILZ) expression in macrophages in the late stage of inflammation, indicating that GILZ might contribute to immunosuppression. However, the conclusions drawn in these two manuscripts regarding the role of endogenous GILZ in macrophages were contradictory.

## GLUCOCORTICOIDS AND THE GLUCOCORTICOID-INDUCED LEUCINE ZIPPER (GILZ)

GILZ was identified in 1997 as a glucocorticoid (GC)-inducible gene from a thymus subtraction library [24] and has been suggested to play a key role in the anti-inflammatory activity of GCs later on [25, 26].

In general, endogenous GCs are involved in feedback mechanisms that terminate inflammation, whereas synthetic GCs are widely used for the treatment of inflammatory diseases. Their anti-inflammatory action is initiated by binding to their intracellular receptor, the GC receptor (GR), which belongs to the superfamily of ligand-modulated transcription factors. Upon ligand binding, the GR/GC complex translocates into the nucleus and promotes the up- or down-regulation of numerous genes. Gene expression can be suppressed either *via* transrepression, i.e. interference of GC/GR monomers with pro-inflammatory transcription factors such as NF-κB or AP-1, or cis-repression, i.e. binding of GC/GR dimers to negative GC response elements (nGRE) located within the promoter regions of target genes. In contrast, gene induction requires the recruitment of GC/GR homodimers to GR response elements (GREs) in the promoters of GC-inducible genes, such as *GILZ* [26–33]. According to a previously widely accepted assumption, transrepression of transcription factors was regarded as the major mechanism by which GCs mediate their anti-inflammatory properties, whereas transactivation was mainly associated with GC side effects. However, a growing number of studies suggests that transactivation by GC/GR dimers is necessary to fully unfold the anti-inflammatory potential of GCs [32, 34–36].

The *GILZ* gene is located on the X-chromosome and three functionally active GREs are present in its promotor region [29, 33]. GC treatment leads to GILZ upregulation in many cell types, including epithelial cells [37], endothelial cells [39] T lymphocytes [24], B cells [40], dendritic cells [41], and macrophages [23, 42, 43]. Other factors that have been shown to induce GILZ expression include IL-10, transforming growth factor (TGF)-β, and hepatocyte growth factor (HGF) [41, 42, 44].

On the molecular level, GILZ facilitates its anti-inflammatory activity mainly by binding to the pro-inflammatory transcription factors nuclear factor (NF)-κB and activator protein (AP)-1, thereby preventing their translocation [42, 45–47]. In addition, GILZ also interferes with mitogen-activated protein kinase (MAPK) signaling, e.g. by binding to Ras/Raf, resulting in the inhibition of downstream MAP kinases, such as extracellular signal-regulated kinase (ERK) [22, 48, 49].

The involvement of GILZ in GC actions has been investigated in various cell types and animal models of inflammatory diseases by overexpression or depletion strategies. Due to its strong upregulation by GCs in the thymus, early reports on GILZ focused on its effects on thymocytes and T-lymphocytes [24]. Analysis of GILZ overexpressing cell lines and cells obtained from transgenic mice which overexpress GILZ in the T cell lineage indicated that GILZ mimics most of the GC effects on thymocyte and T lymphocyte apoptosis [24, 45, 47, 50, 51]. Similar to GC, GILZ overexpression skews T lymphocytes towards a Th2 phenotype [52]. In accordance with a reduced Th1 response, GILZ transgenic mice were less susceptible towards dinitrobenzene sulfonic acid (DNBS)-induced colitis with the degree of inhibition being comparable to dexamethasone treatment. In particular, these mice showed diminished intestinal tissue damage associated with inhibition of NF-κB nuclear translocation and pro-inflammatory cytokine production in CD4^+^ T lymphocytes of the *lamina propria*. In the same study, inhibition of colitis development was also evident when a GILZ fusion protein was administered to DNBS-treated wildtype (WT) animals and IL-10 knockout mice with fully developed colitis [53].

In human monocyte-derived dendritic cells, GILZ overexpression mimics inhibitory GC effects on maturation and activation [41, 54]. Accordingly, downregulation of endogenous GILZ *via* siRNA prevented most GC actions, suggesting that GILZ is a key regulator of dendritic cell functions [41].

In monocytes and macrophages, GILZ is constitutively expressed and can be further upregulated by GCs [42, 55]. siRNA-mediated GILZ silencing prevented the inhibition of LPS-induced cytokine and chemokine production by GCs in human monocytes [55]. In accordance, studies with the macrophage-like cell line THP-1 cells indicated that GILZ overexpression results in reduced expression of macrophage activation markers, chemokine expression, and NF-κB activity upon LPS treatment [42]. In murine macrophages, another GC-inducible anti-inflammatory protein, Annexin-A1 (ANXA1), was shown to participate in GILZ induction upon GC treatment and to require GILZ to exert its anti-inflammatory effects [56].

The LPS resistance being characteristic for the inbred mouse strain SPRET/Ei has been linked to genetic variations causing increased GILZ expression. Macrophages from SPRET/Ei mice showed an attenuated IL-6 and IL-12 production after LPS challenge, whereas GILZ knockdown by siRNA abrogated the effect. In addition, GILZ overexpression in hepatocytes by hydrodynamic plasmid injection protected C57BL/6 mice against endotoxemia, suggesting that higher levels of GILZ correlate with enhanced protection against the lethal effects of LPS [33].

Taken together, these investigations suggested a functional role for GILZ in the innate and adaptive immune response, thereby underlining the contribution of GR transactivation to the anti-inflammatory effects of GCs.

Of note, specific upregulation of GILZ *in vivo* might avoid the metabolic side effects of GC therapy, as GILZ and GCs have opposing effects regarding the differentiation of mesenchymal stem cells (MSCs). Whereas GCs induce adipocyte differentiation and suppress osteoblast formation, GILZ overexpression favors osteogenic differentiation [57, 58].

The availability of GILZ knockout (KO) mice initiated several studies that focused on the consequences of GILZ deficiency. The most obvious and surprising result of GILZ depletion was male infertility due to the influence of GILZ expression on spermatogonia stem cell survival and differentiation [59, 60].

A recently published report suggested a role for GILZ in the suppression of Th17 responses in psoriasis, a disease commonly treated with GCs. GILZ expression was downregulated in skin lesions of psioriasis patients, and its expression negatively correlated with levels of pro-inflammatory mediators typically associated with Th17 responses. Accordingly, topical application of the TLR7 agonist imiquimod, a common murine model for IL23- and IL17-dependent psoriasis, induced a higher degree of inflammation in GILZ deficient mice *via* upregulation of Th17-inducing cytokines by dendritic cells and Th17 proliferation. Of note, delivery of GILZ using a cell-permeable fusion protein efficiently suppressed Th17 expansion [61]. Another study indicated that MSCs from WT, but not from GILZ KO animals, have immunosuppressive potential when injected into mice in a murine model of arthritis (collagen-induced arthritis, CIA). In particular, GILZ expression in MSCs was shown to be required for the generation of IL-10-producing regulatory Th17 cells [62].

Moreover, both total and B cell specific GILZ KO mice were recently reported to develop a non-lethal B lymphocytosis due to enhanced B cell survival. Decreased B cell apoptosis in GILZ depleted mice was associated with increased transcriptional activity of NF-κB and overexpression of BCL2 (B cell lymphoma 2) [40]. These findings suggest that a lack of GILZ expression might contribute to the pathogenesis of B cell disorders.

In other settings, however, GILZ KO mice failed to show phenotypic changes compared to their WT counterparts. Contradictory to a previous report of exacerbation of CIA by siRNA-mediated GILZ silencing [63], GILZ knockout in C57BL/6 mice influenced neither disease severity nor the therapeutic efficacy of GCs in several models, including antigen-induced arthritis (AIA), K/BxN serum-transfer arthritis, CIA, and endotoxemia [64]. In contrast, local induction of GILZ expression by injection of GILZ-adeno-associated virus (GILZ-AAV) into the joints inhibited disease development in the CIA model [64], leading to the conclusion that endogenous GILZ had no effect on inflammatory effector pathways in arthritis, whereas the administration of exogenous GILZ might have some therapeutic value.

## REGULATION AND FUNCTION OF ENDOGENOUS GILZ EXPRESSION IN MACROPHAGES

Although several investigations deal with the pharmacological induction of GILZ, few is known about the regulation of endogenous GILZ during the immune response in the absence of pharmacological intervention. In both epithelial [37] and endothelial cells [38], inflammatory cytokines have been reported to reduce GILZ expression. Also activated macrophages in patients with Crohn's disease or tuberculosis were shown to lack GILZ [42], and patients suffering from chronic rhinosinusitis have reduced GILZ nasal explant expression [65]. Likewise, *GILZ* mRNA levels were decreased in the livers of patients with alcoholic hepatitis [55].

Besides the pronounced expression levels of GILZ mRNA in endothelial cells [38, 66], we previously demonstrated high GILZ expression levels in primary human *in vitro* differentiated and pulmonary macrophages [66]. GILZ levels were diminished in human alveolar macrophages as well as *in vivo* in mouse lungs upon TLR activation. Our study further demonstrated that *GILZ* mRNA was destabilized upon MyD88-mediated TLR activation, since LPS and Pam3CSK4, but not poly(I:C), the ligand for MyD88-independent TLR3, reduced *GILZ* mRNA. Accordingly, MyD88 knockdown abrogated TLR2- and TLR4-facilitated *GILZ* mRNA decay. Downregulation of *GILZ* required the presence of both the *GILZ* 3′-untranslated region and the RNA-binding protein tristetraprolin (TTP), as shown by overexpression and knockdown experiments [66]. TTP also seems to be involved in the TNF-α mediated downregulation of GILZ in the vasculature: TNF-α induced TTP in primary human endothelial cells, and also degenerated inflamed vessels showed higher levels of TTP. In line with these findings, anti-atherogenic laminar shear stress increased GILZ levels and attenuated TTP induction facilitated by TNF-α [38].

Investigations on the functional significance of GILZ downregulation in macrophages using siRNA-mediated GILZ knockdown revealed an enhanced sensitivity towards LPS, as indicated by increased cytokine expression and NF-κB activity [66]. In a recent follow-up study [22], we crossed LysMCre transgenic mice with mice bearing LoxP sites upstream and downstream of *Gilz* exon 6 [59], resulting in a deletion of GILZ in the myeloid lineage. Bone-marrow derived macrophages (BMM) from these conditional GILZ KO mice were used to determine the influence of GILZ on macrophage responses after LPS exposure. In these cells, the activity of the transcription factors NF-κB and AP-1, the phosphorylation of the MAP kinase ERK, as well as the production of TNF-α and IL-1β were significantly enhanced. These data suggest repression of GILZ expression as a regulatory mechanism that might prolong and/or increase macrophage activation.

In order to assess the contribution of endogenous GILZ on macrophage deactivation, we examined its regulation and function in endotoxin-tolerant macrophages. GILZ downregulation upon LPS treatment was indeed completely abolished in LPS-tolerized cells, which was accompanied by a lack of TTP induction. Accordingly, *GILZ* mRNA half-life was increased in LPS-tolerant macrophages. Whereas LPS tolerance resulted in decreased MAPK activation and cytokine production in WT cells, the inflammatory response was rescued in GILZ KO BMM. The differential LPS-response in WT *vs*. GILZ KO BMM was far more pronounced in LPS-tolerance, probably because LPS treatment itself results in diminished GILZ expression in non-tolerant cells.

**Figure 1:**
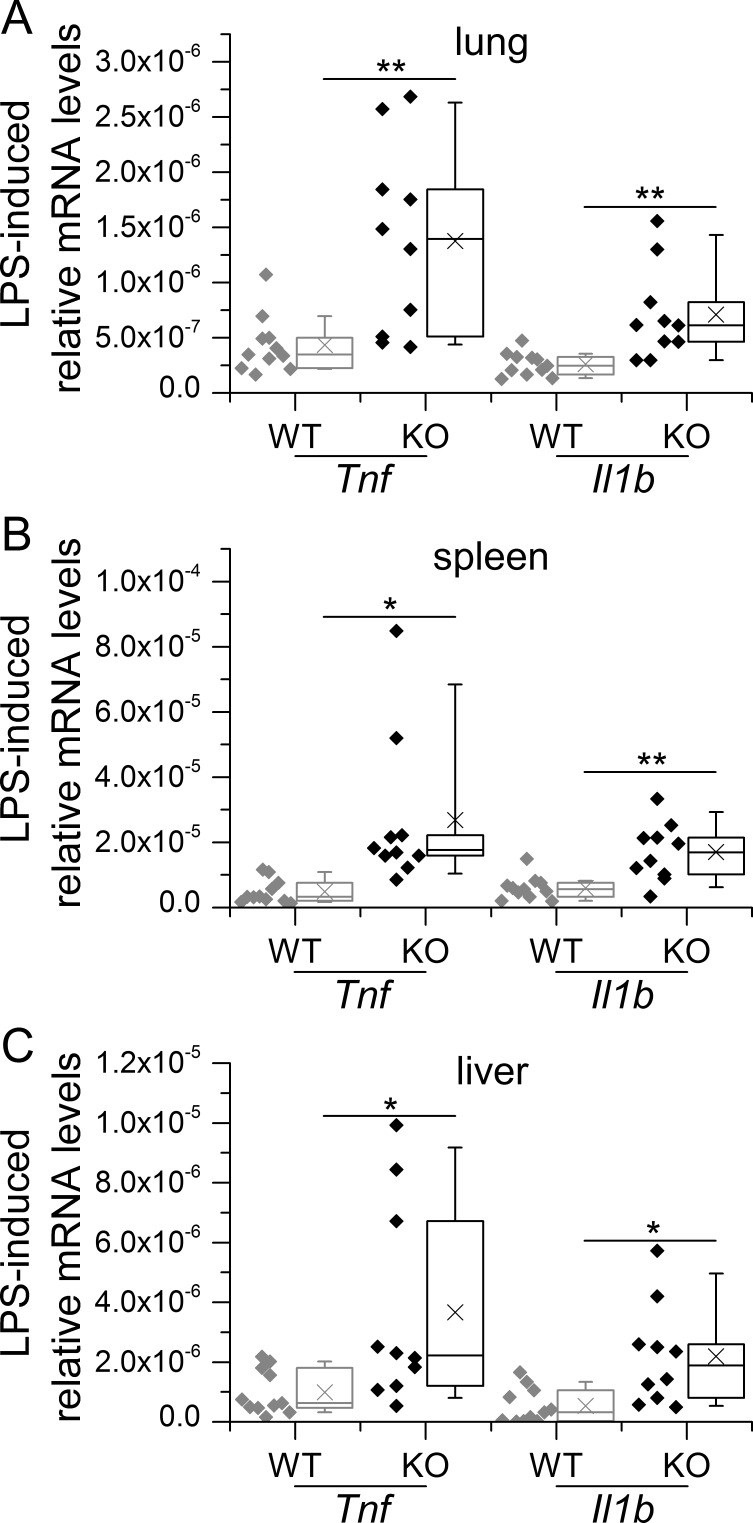
GILZ knockout in myeloid cells abrogates LPS tolerance *in vivo* WT and KO mice were tolerized to LPS by i.p. injections of low-dose LPS (10 μg/mouse) on three consecutive days, followed by a final treatment with high-dose LPS (100 μg/mouse, 4 h) on day 4. Mice were sacrificed and lung **A.**, spleen **B.** and liver **C.** tissues were subjected to qPCR analysis. Data were normalized to *Rn18s* and are shown as individual values and 25^th^/75^th^ percentile boxes with arithmetic medians (cross), geometric medians (line), and 10^th^/90^th^ percentile as whiskers; *n* = 9-12 per treatment group and genotype *p < 0.05, **p < 0.01, ***p < 0.001 (Student's *t*-test). Data were previously presented in [22].

In addition, we also investigated the effects of LPS-tolerance *in vivo*. To this end, WT and conditional GILZ KO mice were subjected to repeated i.p. injections of low-dose LPS, followed by a high dose on day 4. Depleting GILZ in myeloid cells, which are the major source of GILZ in different tissues such as the lung [67], but also considerably contribute to the overall GILZ expression in liver and spleen [22], resulted in a complete abrogation of LPS tolerance *in vivo* (described in [22] and shown in Figure 1).

**Figure 2:**
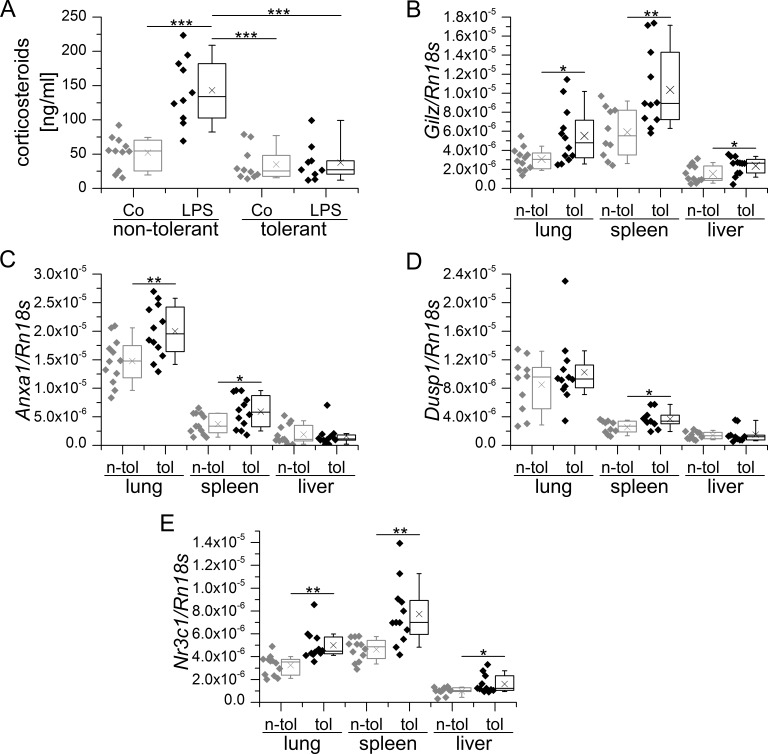
Endogenous corticosteroid levels and GR-mediated transcription in LPS-sensitive and LPS-tolerized WT mice **A.** Mice were either sham-treated (non-tolerant / sensitive) or tolerized towards LPS by repeated low dose LPS injections, followed by either sham- (Co) or high-dose LPS treatment. Serum corticosteroid levels were assessed using a corticosterone ELISA kit (Enzo Life Sciences) according to the manufacturer's instructions. **B.**-**E.**: Tissue samples of sham-treated (n-tol, non-tolerant) or LPS-tolerized (tol) WT mice were analyzed for *Gilz* (B, previously presented in [22]), *Anxa1* **C.**, *Dusp1* **D.** and *Nr3c1* expression. mRNA expression was measured and normalized to *Rn18s*. Data are shown as individual values and 25^th^/75^th^ percentile boxes with arithmetic medians (cross), geometric medians (line), and 10^th^/90^th^ percentile as whiskers; *n* = 10-12 per treatment group. *p < 0.05, **p < 0.01, ***p < 0.001 (A: ANOVA with Bonferroni's post hoc test, B-D: Student's *t*-test).

**Figure 3:**
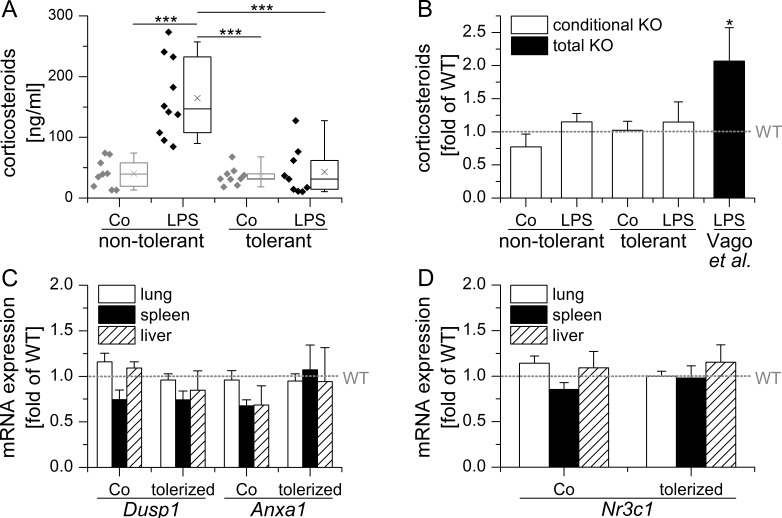
Conditional LysM/Cre mediated GILZ KO does neither influence endogenous GC levels nor GR activity **A.** Conditional GILZ KO mice were either sham-treated (non-tolerant / sensitive) or tolerized towards LPS by repeated low dose LPS injections, followed by either sham- (Co) or high-dose LPS treatment. Serum corticosteroid levels were assessed using a corticosterone ELISA kit (Enzo Life Sciences) according to the manufacturer's instructions. Data are presented as individual values and 25^th^/75^th^ percentile boxes with arithmetic medians (cross), geometric medians (line), and 10^th^/90^th^ percentile as whiskers; *n* = 9-11 per treatment group. ***p < 0.001 (ANOVA with Bonferroni's post hoc test). **B.** Corticosterone serum levels in KO mice are expressed as x-fold of equally treated WT mice + SEM. *p < 0.05 *vs*. WT as reported by Vago et al. [23]. C, D: mRNA expression levels of GC-inducible *Anxa1* and *Dusp1* and the GR *(Nr3c1)* in conditional GILZ KO. mRNA expression was determined in samples previously generated within [22], normalized to *Rn18s* and expressed as x-fold of equally treated WT mice + SEM (*n* = 9-12 per genotype and treatment group). No significant differences *vs.* WT were observed (ANOVA with Bonferroni's post hoc test).

In sepsis and the non-infectious systemic inflammatory response syndrome (SIRS), endogenous corticosteroids are released from the adrenal gland *via* activation of the hypothalamus-pituitary-axis and have been suggested to contribute to the development LPS tolerance [4, 68]. The finding that the GC receptor antagonist RU486 induces a disruption of LPS tolerance in LPS-tolerized mice further supports this assumption [69]. However, endogenous corticosteroid levels were not elevated in LPS tolerance in our hands (Figure 2A). Still, we found an upregulation of GILZ in spleen, lung and liver tissue of LPS-tolerized animals (Figure 2B). Enhanced GC action was further confirmed by the induction of other GC-inducible genes, i.e. *Anxa1* and *Dusp1*, in these tissues (Figure 2C and 2D). As upregulation of GR (*Nr3c1*) expression was evident in all tissues examined, the induction of GR-dependent genes might be due to enhanced sensitivity towards the steady-state levels of endogenous GC (Figure 2E). In contrast, a single high dose of LPS did result neither in the upregulation of GC-inducible genes nor *Nr3c1* in the same tissues (data not shown), although corticosteroid levels were highly increased 4 h after LPS injection (Figure 3A). This observation might be explained by the dominance of pro-inflammatory mediators in early inflammation, which negatively regulate GR function by blocking all levels of the physiological activities of GCs and their receptor [70].

A recently published study by Vago *et al.* [23] investigated the effects of GILZ in natural and GC-driven resolution of LPS-induced pleurisy. In this model, resolution of neutrophilic inflammation took place 48 h after the initiation of inflammation and was paralleled by a mononuclear cell influx. Most interestingly, GILZ expression was increased alongside ANXA1 during the resolution phase, especially in M2 and Mres macrophages. Pretreatment or therapeutic administration of a cell-permeable GILZ fusion protein shortened resolution intervals, thereby suggesting a role for GILZ in resolving inflammation. Surprisingly, the course of inflammation was not modified in total GILZ KO animals. These animals, however, displayed elevated levels of endogenous GC upon LPS injection (Figure 3B), suggesting a deregulation of corsticosteroid homeostasis.

Endogenous GC critically determine the extent of GILZ expression. In adrenalectomized rats lacking endogenous adrenal hormones, GILZ mRNA and protein levels in spleen tissue were significantly reduced [71]. A very recent study demonstrated that *GILZ* mRNA expression in rat adipose tissue follows the circadian rhythm of endogenous GC, although with some evident time delay [72]. Vago *et al.* [23] identified enhanced ANXA1 induction due to elevated endogenous GC levels as a mechanism by which GILZ depletion might be compensated. Indeed, total GILZ KO mice already showed an increased expression of ANXA1 in the absence of any stimulation in pleural cavity cells. When mice were challenged with LPS, ANXA1 was only detectable in its inactive form in cells from WT mice, whereas active ANXA1 was abundant in GILZ KO cells. In accordance, treatment with exogenous GCs resolved inflammation equally well in total GILZ KO and WT mice due to the induction of ANXA1. When ANXA1 was inhibited with a neutralizing antibody, however, GILZ KO mice became refractory to GC treatment.

In contrast to total GILZ KO mice, endogenous GC levels did not differ from those observed in WT animals in our conditional GILZ KO model (Figure 3A and 3B). Accordingly, we detected no differential expression of either *Anxa1*, *Dusp1* or *Nr3c1* in our KO animals when compared to WT mice (Figure 3C and 3D). Of note, we took great care to treat and sacrifice all of our mice at the same time of the day in order to minimize the influence of the circadian GC rhythm.

**Figure 4:**
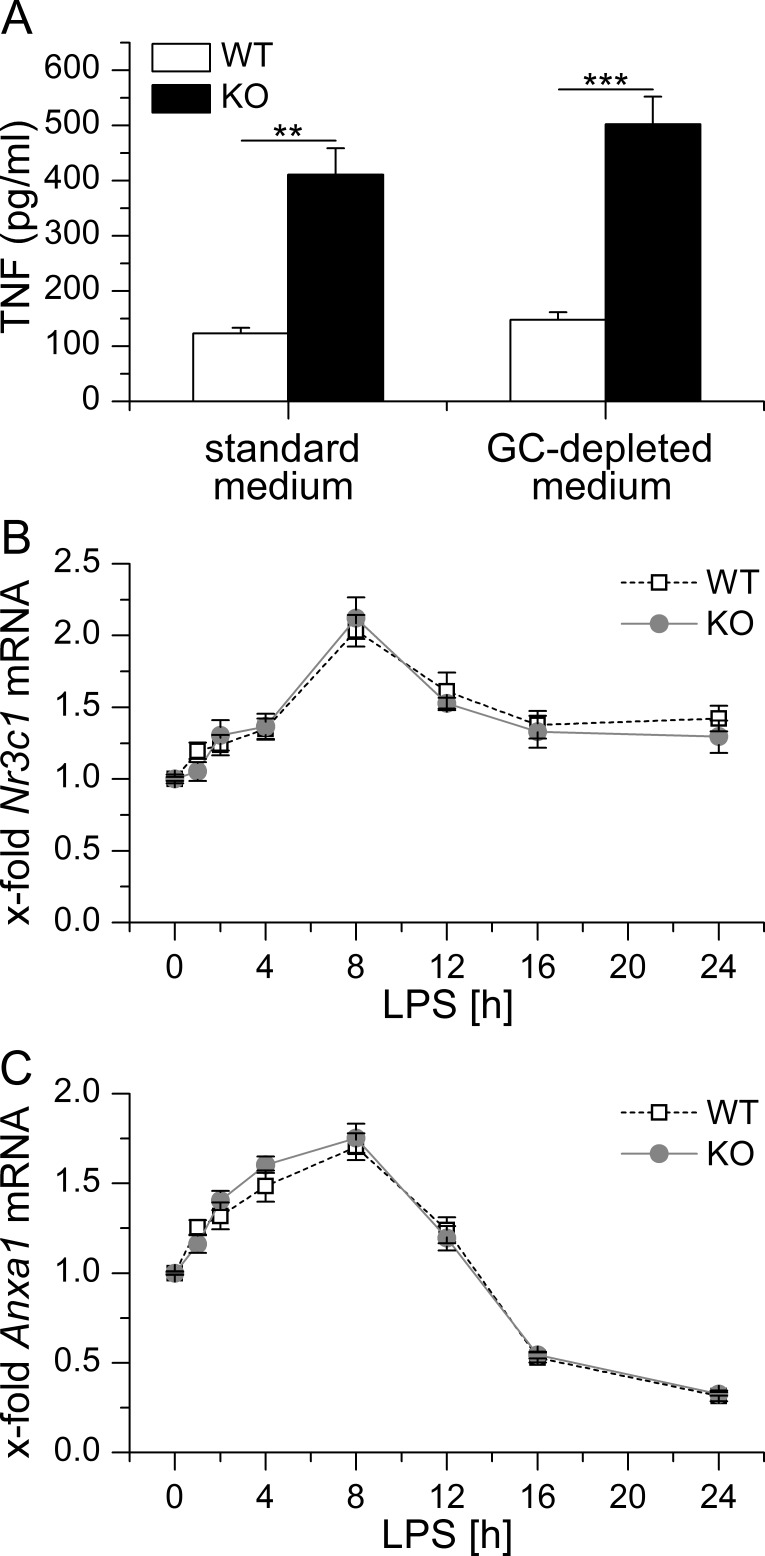
**A.** BMM were either kept in standard or GC-depleted medium, tolerized towards LPS by LPS pretreatment (100 ng/ml) for 24 h and re-challenged with LPS (1 μg/ml) for 4 h. TNF secretion was assessed by bioassay. Data were previously shown in [22]. **B., C.** BMM were treated with 100 ng/ml LPS for up to 24 h. *Nr3c1* **B.** and *Anxa1* **C.** mRNA levels were determined by qPCR, normalized to *Rn18s* and expressed as x-fold of untreated WT BMM (*n* = 4, duplicates). No significant differences in *Nr3c1* or *Anxa1* expression were observed between WT and KO cells (*p* > 0.05, ANOVA with Bonferroni's post hoc test).

In addition, we also checked for a potential influence of GC in our *in vitro* setting. To this end, BMM of WT and KO mice were kept either in standard or GC-depleted medium prior to induction of LPS-tolerance by pretreating the cells for 24 h with LPS. In both standard and GC-depleted media, LPS-tolerant GILZ KO cells released significantly more TNF-α compared with equally treated WT cells, whereas GC depletion had no influence ([22] and Figure 4A). Further analysis of *Anxa1* and *Nr3c1* expression in LPS-treated WT and KO BMM revealed comparable levels of either mRNA (Figure 4B and 4C), suggesting that GC signaling and *Anxa1* expression play no role in our *in vitro* set-up.

Taken together, the studies by Vago *et al*. and ourselves might explain some of the contradictory findings regarding the role of endogenous GILZ. As mentioned above, Ngo *et al*. [64] showed in a previous study that GILZ deficiency did not influence effector pathways of arthritis and was redundant for GC actions. Since total GILZ KO mice were also used in this study, one might speculate that potential effects of GILZ KO could have been compensated by the induction of *Anxa1* or other GC-inducible genes due to elevated endogenous GC levels in these mice as reported by Vago *et al*.. However, it is as yet unknown whether such compensatory mechanisms exist in cell types other than the myeloid lineage and the circumstances that result in differential GC production in WT and total GILZ KO mice are still ill-defined. Thus, the evaluation of endogenous GC levels and the possibility of compensatory mechanisms should be taken into account when using GILZ-depleted animals in future studies.

In conclusion, GILZ might be a potential target for the therapeutic intervention in both inflammatory diseases and immunosuppression. Considering the key role of GILZ in macrophage functions, therapeutic approaches aimed at either up- or downregulating GILZ expression might benefit from the use of macrophage-targeting formulations, such as nanoparticulate delivery systems [73, 74].
